# Solubility Determination, Hansen Solubility Parameters and Thermodynamic Evaluation of Thymoquinone in (Isopropanol + Water) Compositions

**DOI:** 10.3390/molecules26113195

**Published:** 2021-05-26

**Authors:** Mohammed Ghazwani, M. Yasmin Begum, Prawez Alam, Mohammed H. Alqarni, Hasan S. Yusufoglu, Faiyaz Shakeel

**Affiliations:** 1 Department of Pharmaceutics, College of Pharmacy, King Khalid University, Abha 61441, Saudi Arabia; myghazwani@kku.edu.sa (M.G.); ybajen@kku.edu.sa (M.Y.B.); 2 Department of Pharmacognosy, College of Pharmacy, Prince Sattam Bin Abdulaziz University, Al-Kharj 11942, Saudi Arabia; prawez_pharma@yahoo.com (P.A.); m.alqarni@psau.edu.sa (M.H.A.); h.yusufoglu@psau.edu.sa (H.S.Y.); 3 Department of Pharmaceutics, College of Pharmacy, King Saud University, Riyadh 11451, Saudi Arabia

**Keywords:** apparent thermodynamics, Hansen solubility parameters, isopropanol, solubility, thymoquinone

## Abstract

This article studies the solubility, Hansen solubility parameters (HSPs), and thermodynamic behavior of a naturally-derived bioactive thymoquinone (TQ) in different binary combinations of isopropanol (IPA) and water (H_2_O). The mole fraction solubilities (*x*_3_) of TQ in various (IPA + H_2_O) compositions are measured at 298.2–318.2 K and 0.1 MPa. The HSPs of TQ, neat IPA, neat H_2_O, and binary (IPA + H_2_O) compositions free of TQ are also determined. The *x*_3_ data of TQ are regressed by van’t Hoff, Apelblat, Yalkowsky–Roseman, Buchowski–Ksiazczak *λh*, Jouyban–Acree, and Jouyban–Acree–van’t Hoff models. The maximum and minimum *x*_3_ values of TQ are recorded in neat IPA (7.63 × 10^−2^ at 318.2 K) and neat H_2_O (8.25 × 10^−5^ at 298.2 K), respectively. The solubility of TQ is recorded as increasing with the rise in temperature and IPA mass fraction in all (IPA + H_2_O) mixtures, including pure IPA and pure H_2_O. The HSP of TQ is similar to that of pure IPA, suggesting the great potential of IPA in TQ solubilization. The maximum molecular solute-solvent interactions are found in TQ-IPA compared to TQ-H_2_O. A thermodynamic study indicates an endothermic and entropy-driven dissolution of TQ in all (IPA + H_2_O) mixtures, including pure IPA and pure H_2_O.

## 1. Introduction

**Figure 1:**
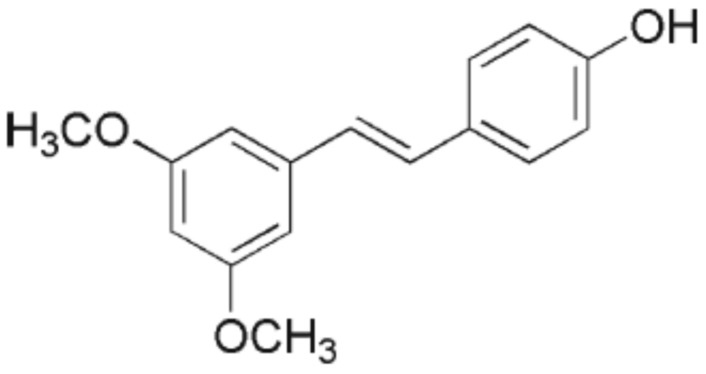
Chemical structure of thymoquinone (TQ).

Thymoquinone (TQ, Figure 1) is a naturally-derived bioactive compound, which is isolated mainly from the essential oils of *Nigella sativa* [1,2]. In the literature, a variety of therapeutic/pharmacological effects of TQ have been reported [3,4,5,6,7]. However, a high amount of TQ is required to produce its therapeutic efficacy, due to its poor water solubility [7,8].

The solubility data of poorly water-soluble naturally-derived bioactive compounds in cosolvent mixtures have a significant role in various industrial processes, such as purification, recrystallization, drug discovery, and formulation development [9,10,11,12,13]. The potential of isopropanol (IPA) has been proved in enhancing the solubility of various poorly soluble bioactive compounds, such as ferulic acid, hesperidin, iminodibenzyl, and quinocetone in the literature [10,14,15,16]. Various formulation strategies, including nanomedicines and advanced drug delivery systems, have been adopted for the solubility/dissolution and bioavailability improvement of TQ in the literature [17,18,19,20,21,22]. The solubility of TQ in neat water (H_2_O) and neat isopropanol (IPA) has been documented well in the literature [7,23,24]. However, the solubility data of TQ in various (IPA + H_2_O) mixtures is not studied and reported in the literature.

Therefore, the solubility data of TQ in various (IPA + H_2_O) mixtures, including pure IPA and pure H_2_O at 298.2–318.2 K and 0.1 MPa, respectively, are studied and reported in this work. The thermodynamic behavior of TQ in various (IPA + H_2_O) compositions, including neat IPA and neat H_2_O, was also evaluated. The solubility parameters for free TQ, neat IPA, neat H_2_O, and various (IPA + H_2_O) mixtures free of TQ were also predicted to correlate with experimental solubility data. The activity coefficients of TQ were also determined to evaluate the TQ-IPA or TQ-H_2_O interactions at the molecular level. The physicochemical data of TQ in various (IPA + H_2_O) combinations reported in this investigation would be applicable in “purification, recrystallization, drug discovery, preformulation studies, formulation development, and preclinical studies” of TQ at laboratory and industrial scale.

## 2. Results and Discussion

### 2.1. TQ Solubility Determination

**Figure 2:**
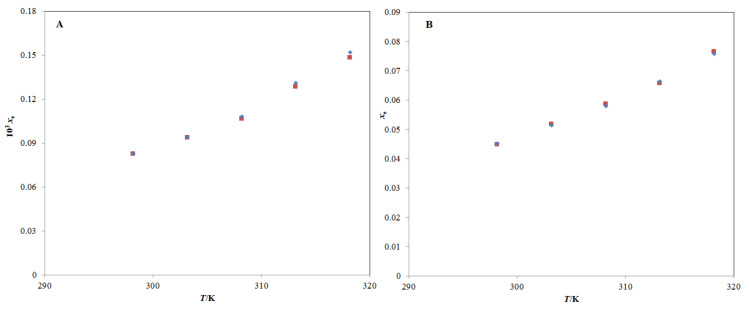
Graphical comparison of TQ solubility in (**A**) neat H_2_O and (**B**) neat IPA with literature values at 298.2–318.2 K; the symbol 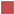 represents the TQ experimental solubility in (**A**) neat H_2_O and (**B**) neat IPA, and the symbol 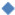 represents the TQ literature solubility values in (**A**) neat H_2_O and (**B**) neat IPA taken from reference [24].

The experimental solubilities of TQ in binary (IPA + H_2_O) mixtures, including pure IPA and pure H_2_O at 298.2–318.2 and 0.1 MPa, respectively, are included in Table 1. The solubility of TQ in pure IPA and pure H_2_O are documented well in the literature [7,23,24]. However, there are no reports on solubility values of TQ in binary (IPA + H_2_O) combinations. Some other cosolvents, such as dimethyl sulfoxide, carbitol, and polyethyleen glycol-400, etc., have been studied to solubilize TQ in our previous publication [24]. In this study, only IPA was studied in the solubilization of TQ in H_2_O. The equilibrium solubility of TQ in neat H_2_O at 298.2 K was reported as 740.63 µg mL^−1^ (equivalent to 7.75 × 10^−5^ in mole fraction) by Salmani et al. [23]. The mole fraction solubility of TQ in neat H_2_O at 298.2 K was determined as 8.27 × 10^−5^ by Ahmad et al. [24]. The mole fraction solubility of TQ in neat H_2_O at 298.2 K was obtained as 8.25 × 10^−3^ in this research (Table 1). The equilibrium solubility of TQ in neat IPA at 298.2 K was reported as 165.54 mg mL^−1^ (equivalent to 4.55 × 10^−2^ in mole fraction) by Kalam et al. [7]. The mole fraction solubility of TQ in neat IPA at 298.2 K was reported as 4.51 × 10^−2^ by Ahmad et al. [24]. The mole fraction solubility of TQ in neat IPA at 298.2 K was recorded as 4.47 × 10^−2^ in this work. The mole fraction solubilities of TQ in pure IPA and pure H_2_O at 298.2–318.2 K have also been reported [24]. The graphical analysis between experimental and literature solubility data of TQ in pure H_2_O and pure IPA at 298.2–318.2 are presented in Figure 2A,B, respectively. 

The results depicted in Figure 2A,B showed an excellent correlation of experimental solubility data of TQ with its literature solubilities in neat H_2_O and neat IPA at 298.2–318.2 K [24]. The root mean square deviation (*RMSD*) between experimental and literature solubility values of TQ in H_2_O and IPA was found to be 1.82% and 0.86%, respectively. These observations indicated that the experimental solubility values of TQ obtained in this research were in good agreement with its literature solubility values [7,23,24]. 

**Figure 3:**
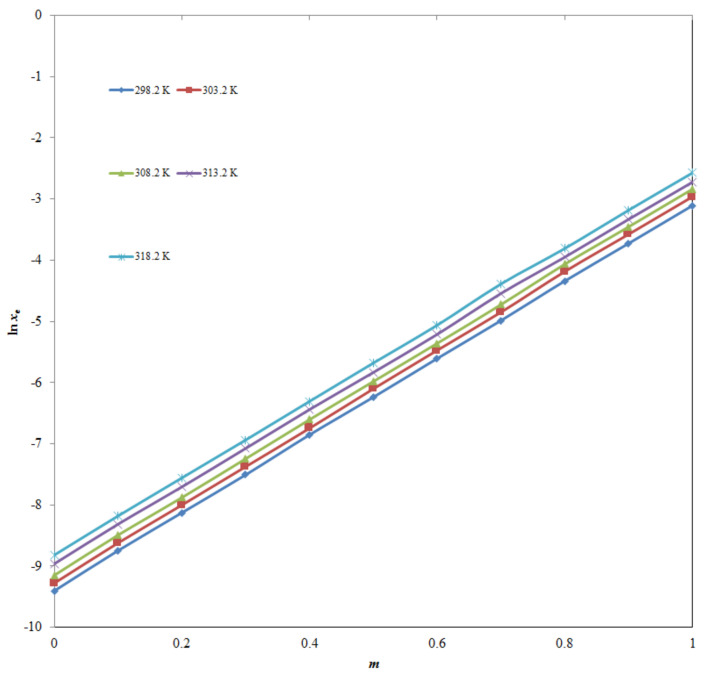
Impact of IPA mass fraction (*m*) on TQ logarithmic solubility at 298.2–318.2 K.

In general, TQ mole fraction solubilities (*x*_3_) were found to be increasing significantly (*p* < 0.05) with the rise in temperature and IPA mass fraction in (IPA + H_2_O) mixtures, and hence, the lowest mole fraction solubility of TQ was recorded in neat H_2_O (*x*_3_ = 8.25 × 10^−5^) at 298.2 K and the highest one was recorded in neat IPA (*x*_3_ = 7.63 × 10^−2^) at 318.2 K. The effect of IPA mass fraction on TQ logarithmic solubilities at 298.2–318.2 K is summarized in Figure 3. According to results depicted in Figure 3, TQ logarithmic solubility was found to be increased significantly with an increase in IPA mass fraction at 298.2–318.2 K (*p* < 0.05). The maximum solubilization/solvation of TQ in IPA could be possible due to lower polarity of IPA, maximum solute-solvent interactions between TQ and IPA, and lower activity coefficient of TQ in IPA compared with water [10]. Accordingly, IPA could be utilized as an efficient cosolvent in TQ solubilization.

### 2.2. Computation of Hansen Solubility Parameters (HSPs)

The HSPs are a very important tool for validating experimental solubility values to find out the best solvent for the solubilization of materials. Therefore, various HSPs for TQ, neat IPA, neat H_2_O, and binary (IPA + H_2_O) compositions free of TQ were determined in this work. The HSPs for binary (IPA + H_2_O) compositions free of TQ at 298.2 K are included in Table 2. The total HSP (*δ*) value of TQ was predicted as 21.00 MPa^1/2^, indicating that TQ is the low-polar bioactive compound. The HSP values for neat IPA (*δ*_1_) and neat H_2_O (*δ*_2_) were predicted as 22.30 and 47.80 MPa^1/2^, respectively. The values of *δ*_mix_ for various (IPA + H_2_O) combinations free of TQ were predicted as 24.85–45.25 MPa^1/2^. The HSP values for neat IPA (*δ*_1_ = 22.30 MPa^1/2^) and neat H_2_O (*δ*_2_ = 47.80 MPa^1/2^) were significantly different (*p* < 0.05). The HSP of TQ was similar to that of neat IPA; hence, the experimental solubilities of TQ were found to be highest in neat IPA compared to its solubilities in neat H_2_O. Overall, the experimental solubility values of TQ in various (IPA + H_2_O) mixtures were in accordance with respective HSPs data.

### 2.3. Determination of Ideal Solubility and Activity Coefficients

The results of ideal solubility (*x*^idl^) values of TQ are included in Table 1. The ideal solubilities of TQ were estimated as 6.16 × 10^−1^–9.92 × 10^−1^ at 298.2–318.2 K. The ideal solubilities of TQ were significantly higher compared to its experimental solubilities in neat IPA, neat H_2_O, and various (IPA + H_2_O) mixtures (*p* < 0.05). The ideal solubilities of TQ were increased significantly with an increase in temperature, which indicated that the dissolution process of TQ was endothermic. The higher ideal solubilities of TQ compared to its experimental solubilities were possible, due to its low melting temperature (352.8 K) [24]. 

The activity coefficient (*γ*_i_) values for TQ in various (IPA + H_2_O) compositions at 298.2–318.2 K are included in Table 3. The *γ*_i_ value of TQ was obtained larger in neat H_2_O at 298.2–323.2 K. However, the *γ*_i_ of TQ was found lowest in neat IPA at 298.2–318.2 K. The larger *γ*_i_ values in water were possible, due to the lowest solubility values of TQ in water. However, the smaller *γ*_i_ values of TQ were due to the highest solubility values of TQ in IPA [10]. It has been reported very well in the literature that low values of *γ*_i_ are associated with maximum solute-solvent interactions at the molecular level [10,24]. The *γ*_i_ values of TQ in IPA were lowest compared to the H_2_O; therefore, the maximum solute-solvent interactions at the molecular level were considered in TQ-IPA compared to the TQ-H_2_O. 

### 2.4. Apparent Thermodynamic Analysis of TQ Solution

The results of various apparent thermodynamic parameters for TQ dissolution in different (IPA + H_2_O) mixtures, including pure IPA and pure H_2_O, are included in Table 4. 

The apparent standard enthalpy (Δ_sol_*H*^0^) values for TQ dissolution in various (IPA + H_2_O) mixtures, including pure IPA and pure H_2_O, were calculated as 20.51–23.47 kJ/mol, suggesting an endothermic dissolution process of TQ in all (IPA + H_2_O) mixtures, including pure IPA and pure H_2_O [25,26]. The apparent standard Gibbs energy (Δ_sol_*G*^0^) values for TQ in binary (IPA + H_2_O) compositions, including pure IPA and pure H_2_O, were estimated as 7.27–23.35 kJ/mol (Table 4). The Δ_sol_*G*^0^ values for TQ were decreased with an increase in IPA mass fraction in all (IPA + H_2_O) compositions. The highest and lowest Δ_sol_*G*^0^ values for TQ were found in pure H_2_O (23.35 kJ/mol) and pure IPA (7.27 kJ/mol), respectively. The highest and lowest Δ_sol_*G*^0^ values for TQ in pure H_2_O and pure IPA were possible due to the lowest and highest solubility values of TQ in pure H_2_O and pure IPA, respectively. The apparent standard entropy (Δ_sol_*S*^0^) values for TQ dissolution in binary (IPA + H_2_O) mixtures, including pure IPA and pure H_2_O, were calculated as 0.36–43.48 J/mol/K, showing an entropy-driven dissolution process of TQ in all (IPA + H_2_O) compositions, including neat IPA and neat H_2_O [25]. Based on these observations and results, the overall TQ dissolution was found to be endothermic and entropy-driven in all (IPA + H_2_O) combinations, including pure IPA and pure H_2_O [25,26].

### 2.5. Modeling of TQ Solubility

The obtained experimental solubilities of TQ were regressed with six different theoretical models, namely, van’t Hoff model (VHM), modified Apelblat model (MAM), Yalkowsky–Roseman model (YRM), Buchowski–Ksiazczak *λh* model (BK*λh*M), Jouyban–Acree model (JAM), and Jouyban–Acree–van’t Hoff (JAVHM) models [26,27,28,29,30,31,32,33,34,35,36]. Results of the VHM model for TQ in binary (IPA + H_2_O) mixtures, including pure IPA and pure H_2_O, are summarized in Table 5. The *RMSD*s for TQ in various (IPA + H_2_O) mixtures, including pure IPA and pure H_2_O, were computed as 0.83–2.09%. An average *RMSD* for the VHM model was calculated as 1.51%. The determination coefficient (*R*^2^) values for VHM were predicted as 0.9921–0.9980. 

**Figure 4:**
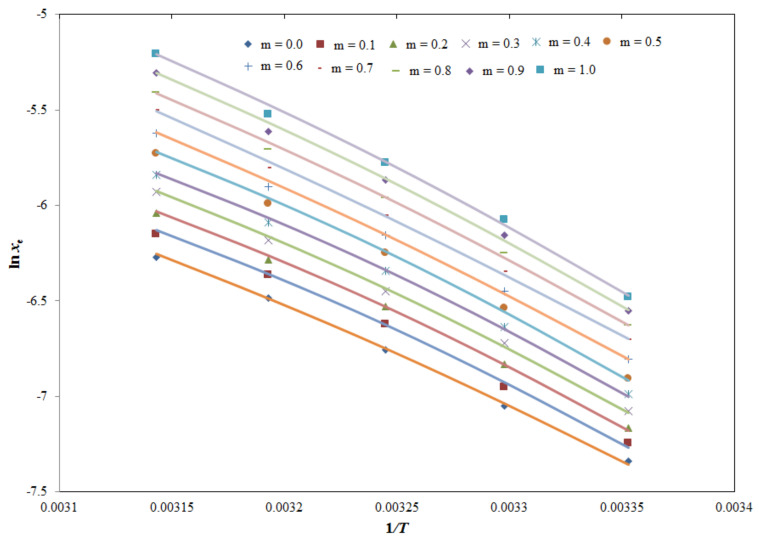
Graphical correlation of TQ solubility with the modified Apelblat model in various (IPA + H_2_O) mixtures at 298.2–318.2 K (the modified Apelblat solubility of TQ is indicated by solid lines, and experimental solubility of TQ is shown by the symbols).

Results of the MAM model for TQ in various (IPA + H_2_O) mixtures, including pure IPA and pure H_2_O, are summarized in Table 6. The graphical comparison between the experimental and MAM solubilities of TQ is shown in Figure 4, which showed an excellent correlation between the experimental and MAM solubility data of TQ. The *RMSD*s for TQ in various (IPA + H_2_O) mixtures, including pure IPA and pure H_2_O, were predicted as 0.51–1.11%. An average *RMSD* for the MAM model was predicted as 0.84%. The *R*^2^ values for the MAM model were estimated as 0.9965–0.9997.

Results of the YRM model for TQ in various (IPA + H_2_O) compositions are summarized in Table 7. The *RMSD*s for TQ in various (IPA + H_2_O) mixtures were predicted as 1.18–3.33%. An average *RMSD* for the YRM model was predicted as 2.02%.

Results of the BK*λh*M model for TQ in binary (IPA + H_2_O) compositions are summarized in Table 8. The *RMSD*s for TQ in various (IPA + H_2_O) compositions were predicted as 1.36–3.24%. An average *RMSD* for the BK*λh*M model was predicted as 2.43%.

Results of the JAM model for TQ in various (IPA + H_2_O) mixtures are summarized in Table 9. An average *RMSD* for the JAM model was predicted as 0.72%.

Results of the JAVHM model for TQ in binary (IPA + H_2_O) compositions are also summarized in Table 9. An average *RMSD* for the JAVHM model was estimated as 0.64%. According to these observations and results, all investigated theoretical models showed low values of *RMSD*, which indicated good solubility correlation for TQ with all six theoretical models evaluated.

## 3. Materials and Methods

### 3.1. Materials

TQ (mass fraction purity = 0.980) and IPA (mass fraction purity = 0.997) were obtained from “Sigma Aldrich (St. Louis, MO, USA)”. Liquid chromatography grade methanol (mass fraction purity = 0.999) was obtained from “Alfa Aesar (Tewksbury, MA, USA)”. The potassium dihydrogen phosphate buffer (10 mM) was prepared in the laboratory. Water (conductivity < 1.0 µS/cm) was obtained from the “Milli-Q Water Purification Unit (Darmstadt, Germany)”. Biological shaker was obtained from “Grant Scientific (Cambridge, UK)”. High-performance liquid chromatography (HPLC) system was from “Waters Corporation (Milford, MA, USA)”. The information about these materials is summarized in Table 10.

### 3.2. Preparation of Solvent Mixtures

All (IPA + H_2_O) solvent mixtures were prepared on mass, using a “Digital Analytical Balance (Mettler Toledo, Greifensee, Switzerland)” with a sensitivity ± 0.10 mg. The mass fraction of IPA for different (IPA + H_2_O) solvent mixtures was varied by 0.10 from 0.10–0.90. Each solvent mixture was obtained in triplicates.

### 3.3. TQ Solubility Evaluation

An isothermal equilibrium method was applied to determine TQ solubility in various (IPA + H_2_O) compositions [*m* = 0.1–0.9; where *m* is the IPA mass fraction in (IPA + H_2_O) compositions, including neat IPA (*m* = 1.0) and neat H_2_O (*m* = 0.0)] [37]. The experiment was performed at 298.2–318.2 K and 0.1 MPa in triplicates. Excess amounts of TQ crystals were dispersed into 2.0 g of each (IPA + H_2_O) mixture, including neat solvents (IPA and H_2_O). Each sample was kept at “OLS 200 Grant Scientific Biological Shaker (Grant Scientific, Cambridge, UK)” after the settings of temperature and rotational speed. The samples were continuously shaken to reach equilibrium. After equilibrium, the samples were taken out from the shaker and centrifuged. The supernatants were carefully withdrawn and diluted with mobile phase and utilized to determine TQ content using the HPLC method at 254 nm, as described in the literature [38]. “Waters HPLC system (Waters Corporation, Milford, MA, USA)” was utilized for TQ analysis. The binary mixture of potassium dihydrogen phosphate buffer (10 mM) and methanol (90:10, *v/v*) was used as the mobile phase. The flow rate of the mobile phase was kept constant at 1.0 mL/min with an isocratic mode. The injection volume for all samples was 20 µL. The column used for TQ analysis was Waters Symmetry-R RP C-18 (150 × 4.6 mm, 5 µm particle size). The TQ content in the above samples was calculated from the previously constructed calibration curve of TQ. The TQ calibration curve was constructed on a mass/mass basis, which was observed as linear in the range of 1–200 µg/g. The *x*_3_ value of TQ was then calculated using Equations (1) and (2) [10,11]:

(1) $$x_{3}=\frac{m_{1}/M_{1}}{m_{1}/M_{1}+m_{2}/M_{2}}$$

(2) $$x_{3}=\frac{m_{1}/M_{1}}{m_{1}/M_{1}+m_{2}/M_{2}+m_{3}/M_{3}}$$

where, *m*_1_ = TQ mass; *m*_2_ = IPA mass; *m*_3_ = H_2_O mass; *M*_1_ = TQ molar mass; *M*_2_ = IPA molar mass, and *M*_3_ = H_2_O molar mass.

### 3.4. Estimation of HSPs

The HSPs of solute, solvent, and cosolvent mixtures are an important tool to predict the best solvent for the solubilization of the solute [39]. Hence, the HSPs for TQ, neat IPA, neat H_2_O, and different (IPA + H_2_O) compositions free of TQ were predicted to compare the experimental solubility values of TQ. The HSP (*δ*) for TQ, neat IPA, and neat H_2_O was obtained by applying Equation (3) [25,39]:

(3) $$\delta^{2}=\delta_{d}^{2}+\delta_{p}^{2}+\delta_{h}^{2}$$

where, “*δ* = total HSP; *δ*_d_ = dispersion HSP; *δ*_p_ = polar HSP and *δ*_h_ = hydrogen-bonded HSP”. The HSPs for TQ, pure IPA, and pure H_2_O were computed by “HSPiP software (version 4.1.07, Louisville, KY, USA)” [39]. However, the HSPs of different (IPA + H_2_O) compositions free of TQ (*δ*_mix_) were obtained using Equation (4) [26,40]:

(4) $$\delta_{\mathrm{mix}}=\propto \delta_{1}+\left(1-\propto \right)\delta_{2}$$

where, *α* = IPA volume fraction in (IPA + H_2_O) combinations; *δ*_1_ = HSP of pure IPA, and *δ*_2_ = HSP of pure H_2_O.

### 3.5. Determination of Ideal Solubility and Activity Coefficients

The *x*^idl^ of TQ at 298.2–318.2 K was calculated by applying Equation (5) [31]:

(5) $$\ln\;x^{\mathrm{idl}}=\frac{-\Delta H_{\mathrm{fus}}\left(T_{\mathrm{fus}}-T\right)}{RT_{\mathrm{fus}}T}+\left(\frac{\Delta C_{p}}{R}\right)[\frac{T_{\mathrm{fus}}-T}{T}+\ln\left(\frac{T}{T_{\mathrm{fus}}}\right)]$$

In which, *T* = absolute temperature; *T*_fus_ = TQ fusion temperature; *R* = universal gas constant; ∆*H*_fus_ = TQ fusion enthalpy, and ∆*C*_p_ = difference in the molar heat capacity of TQ solid state with that of TQ liquid state [9,41]. The quantitative data of *T*_fus_ = 318.52 K, ∆*H*_fus_ = 19.44 kJ/mol, and ∆*C*_p_ = 61.03 J/mol/K were taken from the literature [24]. The *x*^idl^ value for TQ was calculated by applying Equation (5). 

The *γ*_i_ for TQ in binary (IPA + H_2_O) compositions, including pure IPA and pure H_2_O, were obtained by applying the non-random two-liquid (NRTL) model [42]. Based on the principle of solid-liquid equilibrium, the *γ*_i_ can be calculated using the NRTL model with the help of Equation (6) [42,43]:

(6) $$\ln\;\gamma_{i}=\frac{\Delta H_{\mathrm{fus}}}{R}\left(\frac{1}{T_{\mathrm{fus}}}-\frac{1}{T}\right)-\left(\frac{\Delta C_{p}}{R}\right)+\ln\left(\ln\frac{T}{T_{\mathrm{fus}}}-\frac{T}{T_{\mathrm{fus}}}+1\right)]-\ln x_{3}$$

The value of ∆*C*_p_ between the melting and solid states of TQ is very small, and hence, the Equation (6) can be simplified as Equation (7) [43]:

(7) $$\ln\gamma_{i}=\frac{\Delta H_{\mathrm{fus}}}{R}\left(\frac{1}{T_{\mathrm{fus}}}-\frac{1}{T}\right)-\ln x_{3}$$

The interactions between TQ and the solvents or solvent mixtures at the molecular level were evaluated based on the recorded TQ *γ*_i_ data.

### 3.6. Apparent Thermodynamic Analysis of TQ Solution

Apparent thermodynamic analysis of TQ in binary (IPA + H_2_O) compositions, including pure IPA and pure H_2_O, was performed to evaluate TQ dissolution character by applying van’t Hoff and Gibbs Equations. The van’t Hoff Equation was applied to compute the thermodynamic parameters of TQ in various (IPA + H_2_O) compositions at mean harmonic temperature (*T*_hm_) = 308 K at 298.2–318.2 K. The Δ_sol_*H*^0^ value can be obtained using the Equation (8) [41,44]:

(8) ∂ln x3∂1T−1ThmP=−ΔsolH0R

By plotting ln *x*_3_ versus 1T−1Thm, the Δ_sol_*H*^0^ and Δ_sol_*G*^0^ data for TQ were determined using Equations (9) and (10), respectively [45]:

(9) ΔsolH0=−R∂ln x3∂1T−1ThmP

(10) $$\Delta_{\mathrm{sol}}G^{0}=-RT_{\mathrm{hm}}\times \mathrm{intercept}$$

The Δ_sol_*S*^0^ data for TQ in various (IPA + H_2_O) combinations, including neat IPA and neat H_2_O, were computed by applying Equation (11) [41,44,45]:

(11) $$\Delta_{\mathrm{sol}}S^{0}=\frac{\Delta_{\mathrm{sol}}H^{0}-\Delta_{\mathrm{sol}}G^{0}}{T_{\mathrm{hm}}}$$

### 3.7. Solubility Correlation With Theoretical/Computational Models

The experimental solubilities of TQ in binary (IPA + H_2_O) mixtures, including pure IPA and pure H_2_O, were regressed by VHM, MAM, YRM, BK*λh*M, JAM, and JAVHM models [26,27,28,29,30,31,32,33,34,35,36]. 

The VHM model is a semi-predictive model, which can correlate the solubility of solutes at different temperatures at the given set of cosolvent mixtures. Therefore, the VHM solubility (*x*_3_^van’t^) of TQ in different (IPA + H_2_O) mixtures, including pure IPA and pure H_2_O, at different temperatures was computed by applying Equation (12) [26]

(12) $$\ln\;x_{3}^{{\mathrm{van}}^{\prime }t}=a+\frac{b}{T}$$

where, *a* and *b* = model coefficients, and these values were predicted using the least square method. 

The regression between experimental and VHM solubility of TQ was performed using *RMSD* and *R*^2^ values. The *RMSD* of TQ was obtained using its standard equation, reported previously [9]. The MAM model is also a semi-predictive model, which correlates the solubility of solutes at different temperatures at the given set of cosolvent mixtures. Therefore, the MAM solubility (*x*_3_^Apl^) of TQ in binary (IPA + H_2_O) mixtures, including pure IPA and pure H_2_O, was predicted by applying Equation (13) [27,28]:

(13) $$\ln\;x_{3}^{\mathrm{Apl}}=A+\frac{B}{T}+C\ln\left(T\right)$$

where, *A, B*, and *C* = the model coefficients, and these values were predicted using nonlinear multivariate regression analysis of experimental solubilities TQ included in Table 2 [9]. The regression between experimental and MAM solubility of TQ was performed using *RMSD* and *R*^2^. The YRM model is a cosolvency-based model. It is a special case of the JAM model in which adjustable parameters are fixed and equivalent to zero. The logarithmic solubility of the YRM model (log *x*_3_^Yal^) for TQ in various (IPA + H_2_O) compositions was computed by applying Equation (14) [29]:

(14) $$\mathrm{Log}x_{3}^{\mathrm{Yal}}=m_{1}\log x_{1}+m_{2}\log x_{2}$$

where, *x*_1_ = mole fraction solubility of TQ in IPA; *x*_2_ = mole fraction solubility of TQ in H_2_O; *m*_1_ = IPA mass fraction, and *m*_2_ = H_2_O mass fraction.

The BK*λh*M model is based on two parameters *λ* and *h*. It is also a semi-predictive model, which is widely used for the solubility correlation at different temperatures at the given set of cosolvent compositions. The BK*λh*M solubility (*x*_3_^λh^) of TQ was obtained using Equation (15) [30,31]:

(15) $$\ln\;[1+\frac{\lambda \left(1-x_{3}^{\lambda h}\right)}{x_{3}^{\lambda h}}]=\lambda h\;[\frac{1}{T}-\frac{1}{T_{\mathrm{fus}}}]$$

where, *λ* and *h* = the adjustable model parameters of Equation (15). 

The regression between experimental and BK*λh*M solubility of TQ was performed using *RMSD*. The JAM predicts the solubility of solutes in cosolvent mixtures at the given set of temperatures. The JAM solubility (*x*_3_^JA^) of TQ in binary (IPA + H_2_O) composition was predicted using Equation (16) [32,33,34,35,36,46]:

(16) $$\ln\;x_{3}^{\mathrm{JA}}=m_{1}\ln x_{1}+m_{2}\;\ln\;x_{2}+\lceil \frac{m_{1}m_{2}}{T}\overset{2}{\underset{i=0}{\sum }}J_{i}\left(m_{1}-m_{2}\right)\rceil$$

where, *J*_i_ = model coefficient and this value was determined using no-intercept regression analysis, described in the literature [35,47]. The regression between experimental and JAM solubility of TQ was performed using *RMSD*.

The JAVHM model is based on the combination of VHM and JAM models. It also predicts the solubility of solutes in cosolvent mixtures at the given set of temperatures. The JAVHM solubility (*x*_3_^JAVHM^) of TQ in binary (IPA + H_2_O) compositions were predicted using Equation (17) [48]:

(17) $$\ln\;x_{3}^{\mathrm{JAVHM}}=m_{1}\left(A_{1}+\frac{B_{1}}{T}\right)+m_{2}\;\left(A_{2}+\frac{B_{2}}{T}\right)+\left[\frac{m_{1}m_{2}}{T}\;\overset{2}{\underset{i=0}{\sum }}J_{i}\left(m_{1}-m_{2}\right)\right]$$

where, *A_1_*, *B_1_*, *A_2_*, *B_2_*, and *J_i_* = the model coefficients. The regression between experimental and JAVHM solubility of TQ was performed using *RMSD*. 

### 3.8. Statistical Evaluation

Statistical evaluation was performed by applying a Kruskal–Wallis test, followed by Denn’s test using “GraphpadInstat software (Version 9.1.1, San Diego, CA, USA)”. The *p* < 0.05 or equivalent to 0.05 was taken as a significant value.

## 4. Conclusions

In this research, the solubility of a naturally-derived bioactive compound TQ in binary (IPA + H_2_O) mixtures, including pure IPA and pure H_2_O, was evaluated at 298.2–318.2 K and 0.1 MPa. The TQ solubility was found to be increasing significantly with the rise in absolute temperature and IPA mass fraction in all (IPA + H_2_O) mixtures, including pure IPA and pure H_2_O. The experimental solubility values of TQ were regressed well with six different theoretical models, including VHM, MAM, YRM, BK*λh*M, JAM, and JAVHM models. The results of activity coefficient calculations indicated maximum solute-solvent interactions in the TQ-IPA combination compared to the TQ-H_2_O combination. A thermodynamic study suggested an endothermic and entropy-driven dissolution of TQ in all (IPA + H_2_O) mixtures, including pure IPA and pure H_2_O. The obtained solubility data can be utilized for the drug discovery process, formulation development, and preclinical assessment of TQ.

## Figures and Tables

**Table 1:** Experimental solubilities (*x*_e_) of thymoquinone (TQ) in mole fraction in various isopropyl alcohol (IPA) + water (H_2_O) mixtures at 298.2–318.2 K and 0.1 MPa ^a^.

*m*	*x*_e_				
	*T* = 298.2 K	*T* = 303.2 K	*T* = 308.2 K	*T* = 313.2 K	*T* = 318.2 K
0.0	8.25 × 10^−5^	9.35 × 10^−5^	1.06 × 10^−4^	1.28 × 10^−4^	1.48 × 10^−4^
0.1	1.59 × 10^−4^	1.80 × 10^−4^	2.05 × 10^−4^	2.45 × 10^−4^	2.81 × 10^−4^
0.2	2.96 × 10^−4^	3.35 × 10^−4^	3.79 × 10^−4^	4.51 × 10^−4^	5.23 × 10^−4^
0.3	5.50 × 10^−4^	6.27 × 10^−4^	7.15 × 10^−4^	8.47 × 10^−4^	9.91 × 10^−4^
0.4	1.06 × 10^−3^	1.18 × 10^−3^	1.35 × 10^−3^	1.60 × 10^−3^	1.83 × 10^−3^
0.5	1.95 × 10^−3^	2.24 × 10^−3^	2.53 × 10^−3^	2.93 × 10^−3^	3.42 × 10^−3^
0.6	3.68 × 10^−3^	4.20 × 10^−3^	4.71 × 10^−3^	5.46 × 10^−3^	6.34 × 10^−3^
0.7	6.84 × 10^−3^	7.84 × 10^−3^	8.87 × 10^−3^	1.06 × 10^−2^	1.24 × 10^−2^
0.8	1.30 × 10^−2^	1.52 × 10^−2^	1.72 × 10^−2^	1.93 × 10^−2^	2.22 × 10^−2^
0.9	2.41 × 10^−2^	2.80 × 10^−2^	3.16 × 10^−2^	3.58 × 10^−2^	4.15 × 10^−2^
1.0	4.47 × 10^−2^	5.17 × 10^−2^	5.85 × 10^−2^	6.56 × 10^−2^	7.63 × 10^−2^
*x* ^idl^	6.16 × 10^−1^	6.96 × 10^−1^	7.85 × 10^−1^	8.83 × 10^−1^	9.92 × 10^−1^

^a^ The average relative uncertainties *u*_r_ are *u*_r_ (*T*) = 0.012, *u*_r_ (*m*) = 0.010, *u* (*p*) = 0.003 and *u*_r_ (*x*_e_) = 0.014.

**Table 2:** Hansen solubility parameters (*δ*_mix_/MPa^1/2^) for binary (IPA + H_2_O) compositions free of TQ at 298.2 K.

*m*	*δ*_mix_/MPa^1/2^
0.1	45.25
0.2	42.70
0.3	40.15
0.4	37.60
0.5	35.05
0.6	32.50
0.7	29.95
0.8	27.40
0.9	24.85

**Table 3:** Activity coefficients (*γ_i_*) of TQ in binary (IPA + H_2_O) compositions (*m*) at 298.2–318.2 K.

*m*	*γ*_i_				
	*T* = 298.2 K	*T* = 303.2 K	*T* = 308.2 K	*T* = 313.2 K	*T* = 318.2 K
0.0	7350	7383	7349	6877	6702
0.1	3808	3823	3810	3598	3536
0.2	2049	2057	2064	1955	1898
0.3	1103	1099	1094	1042	1021
0.4	573.9	587.2	577.3	552.5	543.7
0.5	310.2	308.0	309.6	301.3	290.6
0.6	164.9	164.2	166.1	161.7	156.6
0.7	88.66	88.06	88.14	82.92	79.84
0.8	46.47	45.30	45.56	45.79	44.71
0.9	25.14	24.66	24.74	24.68	23.90
1.0	13.54	13.34	13.35	13.44	12.99

**Table 4:** Apparent standard enthalpy (Δ_sol_*H*^0^), apparent standard Gibbs energy (Δ_sol_*G*^0^), apparent standard entropy (Δ_sol_*S*^0^), and *R*^2^ values for TQ in binary (IPA + H_2_O) compositions (*m*)^a^.

*m*	Δ_sol_*H*^0^/kJ/mol	Δ_sol_*G*^0^/kJ/mol	Δ_sol_*S*^0^/J/mol/K	*R* ^2^
0.0	23.47	23.35	0.36	0.9923
0.1	22.74	21.68	3.42	0.9942
0.2	22.65	20.10	8.25	0.9967
0.3	22.70	18.50	13.62	0.9965
0.4	22.10	16.87	16.99	0.9935
0.5	21.85	15.27	21.34	0.9962
0.6	21.31	13.67	24.78	0.9957
0.7	23.69	12.02	37.88	0.9932
0.8	20.51	10.41	32.76	0.9978
0.9	21.03	8.84	39.59	0.9980
1.0	20.66	7.27	43.48	0.9975

^a^ Average relative uncertainties are *u*(Δ_sol_*H*^0^) = 0.04, *u*(Δ_sol_*G*^0^) = 0.34 and *u*(Δ_sol_*S*^0^) = 0.67.

**Table 5:** Resulting data of van’t Hoff model for TQ in binary (IPA + H_2_O) compositions (*m*).

*m*	*a*	*b*	*R* ^2^	*RMSD* (%)	Overall *RMSD* (%)
0.0	0.02	–2818.90	0.9921	2.09	
0.1	0.39	–2731.50	0.9940	1.74	
0.2	0.97	–2720.50	0.9926	1.94	
0.3	1.62	–2726.90	0.9964	1.34	
0.4	2.03	–2655.40	0.9933	1.76	
0.5	2.55	–2624.40	0.9961	1.20	1.51
0.6	2.96	–2559.90	0.9956	1.55	
0.7	4.54	–2846.30	0.9930	1.74	
0.8	3.93	–2464.00	0.9979	0.83	
0.9	4.74	–2527.00	0.9980	1.28	
1.0	5.21	–2482.30	0.9975	1.22	

**Table 6:** Resulting data of modified Apelblat model for TQ in binary (IPA + H_2_O) compositions (*m*).

*m*	*A*	*B*	*C*	*R* ^2^	*RMSD* (%)	Overall *RMSD* (%)
0.0	−423.43	16,530.02	62.93	0.9967	1.10	
0.1	−331.45	12,432.20	49.31	0.9970	0.98	
0.2	−446.40	17,720.89	66.49	0.9980	0.80	
0.3	−273.39	9840.95	40.87	0.9984	0.75	
0.4	−325.33	12,303.02	48.65	0.9965	1.11	
0.5	−353.02	13,623.04	52.84	0.9997	0.51	0.84
0.6	−361.45	14,091.46	54.16	0.9995	0.58	
0.7	−455.99	18,196.27	68.44	0.9982	0.89	
0.8	−47.03	−131.00	7.57	0.9977	0.91	
0.9	−160.29	5017.23	24.52	0.9987	0.76	
1.0	−156.94	4929.95	24.09	0.9966	0.85	

**Table 7:** Resulting data of Yalkowsky–Roseman model for TQ in binary (IPA + H_2_O) compositions (*m*) at 298.2–318.2 K.

*m*	Log *x*^Yal^					*RMSD* (%)	Overall *RMSD* (%)
	*T* = 298.2 K	*T* = 303.2 K	*T* = 308.2 K	*T* = 313.2 K	*T* = 318.2 K		
0.1	−3.81	−3.75	−3.70	−3.62	−3.55	2.40	
0.2	−3.53	−3.48	−3.42	−3.35	−3.28	1.29	
0.3	−3.26	−3.20	−3.15	−3.07	−3.01	1.18	
0.4	−2.98	−2.93	−2.87	−2.80	−2.74	2.62	
0.5	−2.71	−2.65	−2.60	−2.53	−2.47	1.71	2.02
0.6	−2.44	−2.38	−2.32	−2.26	−2.20	1.30	
0.7	−2.16	−2.10	−2.05	−1.99	−1.93	3.33	
0.8	−1.89	−1.83	−1.78	−1.72	−1.65	2.87	
0.9	−1.62	−1.56	−1.50	−1.45	−1.38	1.53	

**Table 8:** The Buchowski–Ksiazczak *λh* model parameters (*λ* and *h*) and *RMSD* values for TQ in various (IPA + H_2_O) compositions.

Samples	*λ*	*h*	*RMSD* (%)	Overall *RMSD* (%)
0.1	7.17	380.55	2.59	
0.2	6.56	414.57	2.42	
0.3	5.93	459.14	2.89	
0.4	5.30	500.45	1.98	
0.5	4.68	560.08	1.36	2.43
0.6	4.06	629.23	3.12	
0.7	3.39	838.70	3.24	
0.8	2.80	878.21	1.68	
0.9	2.18	2527.00	2.67	

**Table 9:** Resulting data of Jouyban–Acree and Jouyban–Acree–van’t Hoff models for TQ in (IPA + H_2_O) mixtures.

System	Jouyban–Acree	Jouyban–Acree-van’t Hoff
		*A*_1_ 5.21 *B*_1_ –2482.30 *A*_2_ 0.02 *B*_2_ –2818.90 *J*_i_ 67.38 0.64
(IPA + H_2_O)	*J*_i_ 73.49	
	
	
*RMSD* (%)	0.72	

**Table 10:** List of materials used.

Material		Molecular Formula	Molar Mass (g/mole)	CAS Registry No.	Purification Method	Mass Fraction Purity	Analysis Method	Source
TQ		C_10_H_12_O_2_	164.20	490–91-5	None	0.980	HPLC	Sigma Aldrich
IPA		C_3_H_8_O	60.10	67–63-0	None	0.997	GC	Sigma Aldrich
H_2_O		H_2_O	18.07	7732–18-5	None	-	-	Milli-Q

TQ, thymoquinone; IPA, isopropyl alcohol; H_2_O, water; HPLC, high-performance liquid chromatography; GC, gas chromatography.

## References

[1] Pagola S., Benavente A., Raschi A., Romano E., Molina M.A.A., Stephens P.W. (2003). Crystal structure determination of thymoquinone by high-resolution X-ray powder diffraction. AAPS PharmSciTech.

[2] Sayed M.D. (1980). Traditional medicine in health care. J. Ethnopharmacol..

[3] Ahmad A., Alkharfy K.M., Khan R.M.A. (2013). Effects of selected bioactive natural products on the vasculature. J. Cardiovasc. Pharmacol..

[4] Farkhondeh T., Samarghandian S., Shahri A.M.P., Samini F. (2018). The neuroprotective effects of thymoquinone: A review. Dose Resp..

[5] Al-Logmani A., Zari T. (2011). Long-term effects of *Nigella sativa* L. oil on some physiological parameters in normal and streptozotocin-induced diabetic rats. J. Diab. Mell..

[6] Woo C.C., Kumar A.P., Sethi G., Tan K.H. (2012). Thymoquinone: Potential cure for inflammatory disorders and cancer. Biochem. Pharmacol..

[7] Kalam M.A., Raish M., Ahmed A., Alkharfy K.M., Mohsin K., Alshamsan A., Al-Jenoobi F.I., Al-Mohizea A.M., Shakeel F. (2017). Oral bioavailability enhancement and hepatoprotective effects of thymoquinone by self-nanoemulsifying drug delivery system. Mater. Sci. Eng. C.

[8] Elmowafy M., Samy A., Raslan M.A., Salama A., Said R.A., Abdelaziz A.E., El-Eraky W., El-Awdan S., Vitala T. (2016). Enhancement of bioavailability and pharmacokinetic effects of thymoquinone via nanostructured lipid carrier (NLC) formulation. AAPS PharmSciTech.

[9] Shakeel F., Haq N., Siddiqui N.A. (2019). Thermodynamic solubility and solvation behavior of ferulic acid in different (PEG-400 + water) binary solvent mixtures. Drug Dev. Ind. Pharm..

[10] Haq N., Siddiqui N.A., Shakeel F. (2017). Solubility and molecular interactions of ferulic acid in various (isopropanol + water) mixtures. J. Pharm. Pharmacol..

[11] Shakeel F., Haq N., Alanazi F.K., Alanazi S.A., Alsarra I.A. (2020). Solubility of sinapic acid in various (carbitol + water) systems: Computational modeling and solution thermodynamics. J. Therm. Anal. Calorim..

[12] Ha E.S., Park H., Lee S.K., Sim W.Y., Jeong J.S., Kim M.S. (2020). Equilibrium solubility and modeling of trans-resveratrol in dichloromethane and primary alcohol solvent mixtures at different temperatures. J. Mol. Liq..

[13] Talebi J., Saadatjou N., Farajtabar A. (2020). Hesperetin solubility in aqueous cos-solvent mixtures of methanol and ethanol: Solute descriptors, solvent effect and preferential solvation analysis. J. Sol. Chem..

[14] Xu R., Cong Y., Zheng M., Chen G., Zhao H. (2018). Solubility and modeling of hesperidin in cosolvent mixtures of ethanol, isopropanol, propylene glycol, and n-propanol + water. J. Chem. Eng. Data.

[15] Li X., Liu Y., Farajtabar A., Ding G., Chen G., Zhao H. (2019). Equilibrium solubility determination, modeling and preferential solvation of bioactive iminodibenzyl in aqueous co-solvent mixtures at various temperatures. J. Chem. Thermodyn..

[16] Li W., Zhang N., Farajtabar A., Feng X., Chen G., Li X., Zhao H. (2019). Equilibrium solubility evaluation and thermodynamic analysis of quinocetone in aqueous co-solvent solutions of ethanol, isopropanol, dimethyl sulfoxide and N,N-diemthylformamide. J. Chem. Thermodyn..

[17] Schneider-Stock R., Fakhoury I.H., Zaki A.M., El-Baba1 C.O., Gali-Muhtasib H.U. (2014). Thymoquinone: Fifty years of success in the battle against cancer models. Drug Discov. Today.

[18] Odeh F., Ismail S.I., Abu-Dahab R., Mahmoud I.S., Al-Bawab A. (2012). Thymoquinone in liposomes: A study of loading efficiency and biological activity towards breast cancer. Drug Deliv..

[19] Tubesha Z., Imam M.U., Mahmud R., Ismail M. (2013). Study on the potential toxicity of a thymoquinone-rich fraction nanoemulsion in Sprague Dawley rats. Molecules.

[20] Ravindran J., Nair H.B., Sung B., Prasad S., Tekmal R.R., Aggarwal B.B. (2010). Thymoquinone poly (lactide-co-glycolide) nanoparticles exhibit enhanced anti-proliferative, anti-inflammatory and chemosensitization potential. Biochem. Pharmacol..

[21] Alam S., Khan Z.I., Mustafa G., Kumar M., Islam F., Bhatnagar A., Ahmad F.J. (2012). Development and evaluation of thymoquinone-encapsulated chitosan nanoparticles for nose-to-brain targeting: A pharmacoscintigraphic study. Int. J. Nanomed..

[22] Ramachandran S., Thangarajan S. (2018). Thymoquinone loaded solid lipid nanoparticles counteracts 3-nitropionic acid induced motor impairments and neuroinflammation in rat model of Huntington’s disease. Metab. Brain Dis..

[23] Salmani J.M.M., Asghar S., Lv H., Zhou J. (2014). Aqueous solubility and degradation kinetics of the phytochemical anticancer thymoquinone; probing the effects of solvents, pH and light. Molecules.

[24] Ahmad A., Raish M., Alkharfy K.M., Alsarra I.A., Khan A., Ahad A., Jan B.L., Shakeel F. (2018). Solubility, solubility parameters and solution thermodynamics of thymoquinone in different mono solvents. J. Mol. Liq..

[25] Kalam M.A., Alshehri S., Alshamsan A., Alkholief M., Ali R., Shakeel F. (2019). Solubility measurement, Hansen solubility parameters and solution thermodynamics of gemfibrozil in different pharmaceutically used solvents. Drug Dev. Ind. Pharm..

[26] Shakeel F., Haq N., Alsarra I.A., Alshehri S. (2020). Solubility, Hansen solubility parameters and thermodynamic behavior of emtricitabine in various (polyethylene glycol-400 + water) mixtures: Computational modeling and thermodynamics. Molecules.

[27] Apelblat A., Manzurola E. (1999). Solubilities of o-acetylsalicylic, 4-aminosalicylic, 3,5-dinitrosalicylic and p-toluic acid and magnesium-DL-aspartate in water from T = (278–348) K. J. Chem. Thermodyn..

[28] Manzurola E., Apelblat A. (2002). Solubilities of L-glutamic acid, 3-nitrobenzoic acid, acetylsalicylic, p-toluic acid, calcium-L-lactate, calcium gluconate, magnesium-DL-aspartate, and magnesium-L-lactate in water. J. Chem. Thermodyn..

[29] Yalkowsky S.H., Roseman T.J., Yalkowsky S.H. (1981). Solubilization of drugs by cosolvents. Techniques of Solubilization of Drugs.

[30] Ksiazczak A., Moorthi K., Nagata I. (1994). Solid-solid transition and solubility of even n-alkanes. Fluid Phase Equilib..

[31] Tong Y., Wang Z., Yang E., Pan B., Jiang J., Dang P., Wei H. (2016). Determination and correlation of solubility and solution thermodynamics of ethenzamide in different pure solvents. Fluid Phase Equilib..

[32] Jouyban A. (2008). Review of the cosolvency models for predicting solubility of drugs in water-cosolvent mixtures. J. Pharm. Pharm. Sci..

[33] Sotomayor R.G., Holguín A.R., Romdhani A., Martinez F., Jouyban A. (2013). Solution thermodynamics of piroxicam in some ethanol + water mixtures and correlation with the Jouyban–Acree model. J. Sol. Chem..

[34] Babaei M., Shayanfar A., Rahimpour E., Barzegar-Jalali M., Martinez F., Jouyban A. (2019). Solubility of bosentan in {propylene glycol + water} mixtures at various temperatures: Experimental data and mathematical modeling. Phys. Chem. Liq..

[35] Jouyban A., Chan H.K., Chew N.Y.K., Khoubnasabjafari M., Acree W.E. (2006). Solubility prediction of paracetamol in binary and ternary solvent mixtures using Jouyban-Acree model. Chem. Pharm. Bull..

[36] Jouyban A., Acree W.E. (2006). In silico prediction of drug solubility in water-ethanol mixtures using Jouyban-Acree model. J. Pharm. Pharm. Sci..

[37] Higuchi T., Connors K.A. (1965). Phase-solubility techniques. Adv. Anal. Chem. Instr..

[38] Alkharfy K.M., Ahmad A., Khan R.M., Al-Asmari M. (2013). High-performance liquid chromatography of thymoquinone in rabbit plasma and its application to pharmacokinetics. J. Liq. Chromatogr. Relat. Technol..

[39] Zhu Q.N., Wang Q., Hu Y.B., Abliz X. (2019). Practical determination of the solubility parameters of 1-alkyl-3-methylimidazolium bromide ([CnC1im]Br, n = 5, 6, 7, 8) ionic liquids by inverse gas chromatography and the Hansen solubility parameter. Molecules.

[40] Wan Y., He H., Huang Z., Zhang P., Sha J., Li T., Ren B. (2020). Solubility, thermodynamic modeling and Hansen solubility parameter of 5-norbornene-2,3-dicarboximide in three binary solvents (methanol, ethanol, ethyl acetate + DMF) from 278.15 K to 323.15 K. J. Mol. Liq..

[41] Ruidiaz M.A., Delgado D.R., Martínez F., Marcus Y. (2010). Solubility and preferential solvation of indomethacin in 1,4-dioxane + water solvent mixtures. Fluid Phase Equilib..

[42] Wang J., Xie C., Yin Q., Tao L., Lv J., Wang Y., He F., Hao H. (2016). Measurement and correlation of solubility of cefmenoxime hydrochloride in pure solvents and binary solvent mixtures. J. Chem. Thermodyn..

[43] Zaho K., Yang P., Du S., Li K., Li X., Li Z., Liu Y., Lin L., Hou B. (2016). Measurement and correlation of solubility and thermodynamics of mixing of 4-aminobutyric acid in mono-solvents and binary solvent mixtures. J. Chem. Thermodyn..

[44] Holguín A.R., Rodríguez G.A., Cristancho D.M., Delgado D.R., Martinez F. (2012). Solution thermodynamics of indomethacin in propylene glycol + water mixtures. Fluid Phase Equilib..

[45] Krug R.R., Hunter W.G., Grieger R.A. (1976). Enthalpy-entropy compensation. 2. Separation of the chemical from the statistic effect. J. Phys. Chem..

[46] Khoubnasabjafari M., Shayanfar A., Martínez F., Acree W.E., Jouyban A. (2016). Generally trained models to predict solubility of drugs in carbitol + water mixtures at various temperatures. J. Mol. Liq..

[47] Jouyban A., Acree W.E. (2018). Mathematical derivation of the Jouyban-Acree model to represent solute solubility data in mixed solvents at various temperatures. J. Chem. Eng. Data.

[48] Jouyban-Gharamaleki A., Hanaee J. (1997). A novel method for improvement of predictability of the CNIBS/R-K equation. Int. J. Pharm..

